# A qualitative study on the experiences of women undergoing surgery for developmental breast asymmetry

**DOI:** 10.1177/17455057241274901

**Published:** 2024-09-05

**Authors:** Lorraine Kit Ying Ho, Sahima Jafari, Tamara Crittenden, Phillipa van Essen, Andrea Smallman, Nicola R. Dean

**Affiliations:** 1 College of Medicine and Public Health, Flinders University, Bedford Park, SA, Australia; 2 Department of Plastic and Reconstructive Surgery, Flinders Medical Centre, Bedford Park, SA, Australia

**Keywords:** developmental breast asymmetry, breast reconstruction surgery, qualitative study

## Abstract

**Background:**

Developmental breast asymmetry (DBA) is a largely underreported condition where the natural growth of one breast is smaller than the other. While some degree of asymmetry or difference in size and shape is present in most women, DBA can result in more profound differences that can impact a woman’s psychosocial well-being.

**Objectives:**

This study aims to better understand the experiences of women living with DBA, their experiences seeking treatment, and their reconstructive surgical journey and outcomes.

**Design:**

This was a qualitative study involving in-depth, one-on-one semi-structured interviews with women diagnosed with DBA.

**Methods:**

Participants were women seeking treatment for DBA through the Plastic and Reconstructive Surgery Unit at Flinders Medical Centre, a tertiary healthcare centre in Adelaide, South Australia. Interviews were recorded digitally, transcribed verbatim and analysed thematically.

**Results:**

Fourteen interviews were conducted with 14 women; 13 women had completed their reconstruction and 1 was undergoing reconstruction at the time of their interview. Interviews highlighted the significant psychosocial impact of DBA, the different experiences in seeking help for DBA, the information received or lack thereof, the need for medical and social support throughout the surgical process, and the varied satisfaction with surgical outcomes.

**Conclusion:**

This study highlighted the subjective experiences of women who have grown up with DBA, improving our understanding of the significant psychosocial impact of DBA. Not all participants experienced post-operative improvements in psychosocial well-being due to surgical complications or unmet expectations. This study also demonstrated the need to raise awareness about DBA and the importance of additional medical and social support for women throughout their surgical journey.

## Introduction

The aesthetic appearance of female breasts is often defined by parameters such as breast shape, symmetry, size, volume, nipple location, areola shape and overall appearance.^1^ Differences in these parameters can have important implications for a woman’s sense of femininity, psychosocial well-being and sexual well-being.^2^ However, differences in breast shape and size are common. Clinicians often describe breasts as ‘sisters, not twins’, with 88% of women having some degree of natural asymmetry.^3,4^ Currently, there are no specific measurement criteria to define breast asymmetry; however, several parameters (including breast shape, size, volume and overall appearance) have been used to guide the recognition of clinically significant breast asymmetry.^1^ Recent literature shows that 92% of women have asymmetry in at least two of these parameters and 72% in at least three.^5^

Developmental breast asymmetry (DBA) often becomes prominent during adolescence, a period when women are most critical about their bodies, especially in comparison to others.^2,6^ For these women, having DBA and feeling ‘abnormal’ can be a source of distress and shame. Symmetrisation or reconstruction surgery can help to correct DBA, which in turn may allow women to feel ‘normal’.^6^ Surgery can involve breast reduction, breast augmentation with tissue expanders and implants, as well as mastopexy. Several studies have demonstrated post-operative improvement in psychosocial well-being using ad hoc patient satisfaction questionnaires or surveys.^1,7,8^

Given the profound psychosocial impacts that DBA can have, it is crucial to better understand the experiences of women who have grown up with DBA and how reconstruction surgery has impacted their lives. This can help shape the best approach for women undergoing reconstruction surgery in the future. However, there are limited qualitative studies that have comprehensively evaluated the health impact of DBA and outcomes of reconstruction surgery in affected women. Other studies have provided qualitative insight only into cosmetic breast surgery and reconstructive surgery for breast cancer patients and not DBA.^9,10^ One report by NiMhurchadha et al. has delved specifically into DBA, its impacts and surgical reconstruction outcomes using a qualitative approach. They found that women with DBA wanted ‘normal’ breasts, feared people knowing about their DBA and had improved confidence post-operatively.^6^ However, they did not explore how women sought treatment for DBA, the information and support provided around surgery, and the subjective experiences of the reconstruction journey.

While patient satisfaction questionnaires and surveys can provide valuable data on post-operative outcomes, they do not explore the reasons why a patient might feel satisfied or dissatisfied. For plastic surgery, exploring subjective outcomes and understanding the motivations and experiences of patients can provide powerful insight.^11^ This can help facilitate the delivery of patient-centred care. Through a qualitative approach, this study aims to better understand the subjective experiences of women who have grown up with DBA, their experience of seeking and undergoing breast symmetrisation surgery in Australia, and their surgical outcomes.

## Methods

### Research design

This was a qualitative cohort study using semi-structured interviews to explore participants’ experiences growing up with DBA, going through the process of symmetrisation surgery, and the impact of surgery on their physical and psychosocial well-being.^12^

### Participants

Women over the age of 18 years who had either undergone or were undergoing breast symmetrisation surgery for the correction of DBA at Flinders Medical Centre between 2008 and 2020 in Adelaide, Australia were eligible to be included. Women below 18 years of age were excluded from the study. A letter of invitation and participation information sheet were posted to eligible participants. Women were then followed up with a phone call to determine if they wished to participate in the study. Participants who agreed were contacted again to arrange an interview. Fourteen interviews with 14 women were completed between March 2022 and July 2022. The average age of the participants at the time of surgery was 22.5 years (range 17–35 years). Thirteen of the 14 women had completed their reconstruction at the time of their interview, and 1 was still undergoing reconstruction. Amongst the 13 women who completed their reconstruction, the average length of time between their reconstruction and time of their interview was 8 years (range 2–18 years). Surgical procedures that participants underwent included tissue expanders with subsequent insertion of implants, breast reduction and mastopexy, or a combination of procedures. Interviews were on average 71 min in duration (range 47–93 min).

### Data collection

A semi-structured interview guide was developed based on clinical experience of the study team to allow for in-depth one-on-one interviews (Supplemental Appendix 1). The interviews were conducted either face-to-face in private meeting rooms, or online via Zoom (Zoom Video Communications, San Jose, USA). During the study, Coronavirus disease (COVID-19) restrictions regarding social distancing and entry to hospitals made face-to-face interviews impossible for some participants and online interviews were conducted. Recent evidence has supported the use of online interviews as a practical alternative in qualitative research, without compromise of interview data.^13–15^ Moreover, it was felt that there was no difference in building a good rapport and interview quality between participants interviewed online and those interviewed in person – they were all equally engaged and keen to share their experiences. Interviews were approximately 1 h and were conducted using an interview question guide (Supplemental Appendix 1) by two of the investigators with no relationship to the participants. All interviews were audio-recorded, transcribed verbatim and de-identified.

### Data analysis

Braun and Clarke’s approach to reflexive thematic analysis was used in this study.^12^ Inductive thematic analysis was conducted using NVivo Mac Release 1.7.1 (QSR International, Denver, CO, USA). The first six transcripts were initially inductively coded independently by researchers (LKH and SJ) using a ‘line-by-line’ approach where codes were derived directly from the text rather than being pre-formed.^12,16^ Codes from the interviews were then cross-checked, discussed and confirmed. The remaining eight interviews were then co-coded by researchers LKH and SJ using the initial codes. These were further refined as data analysis progressed. Participants’ quotes and the respective codes were copied into a Microsoft Excel Spreadsheet Version 16.73 (Microsoft Corporation, Redmond, WA, USA) to consolidate the codes. Consolidation helped to group codes that were similar into sub-themes (e.g. not having the right bra), and from there, sub-themes were grouped into main themes (e.g. negative impact of DBA on quality of life). Results from the data analysis were reviewed by members of the research team throughout the study to ensure rigour. Interviews continued until there were no more refinements or developments made to the codes and the researchers felt confident that theoretical saturation had been reached.^17^ The Standards for Reporting Qualitative Research Checklist was used in the design and reporting of this study.

## Results

**Figure 1.**
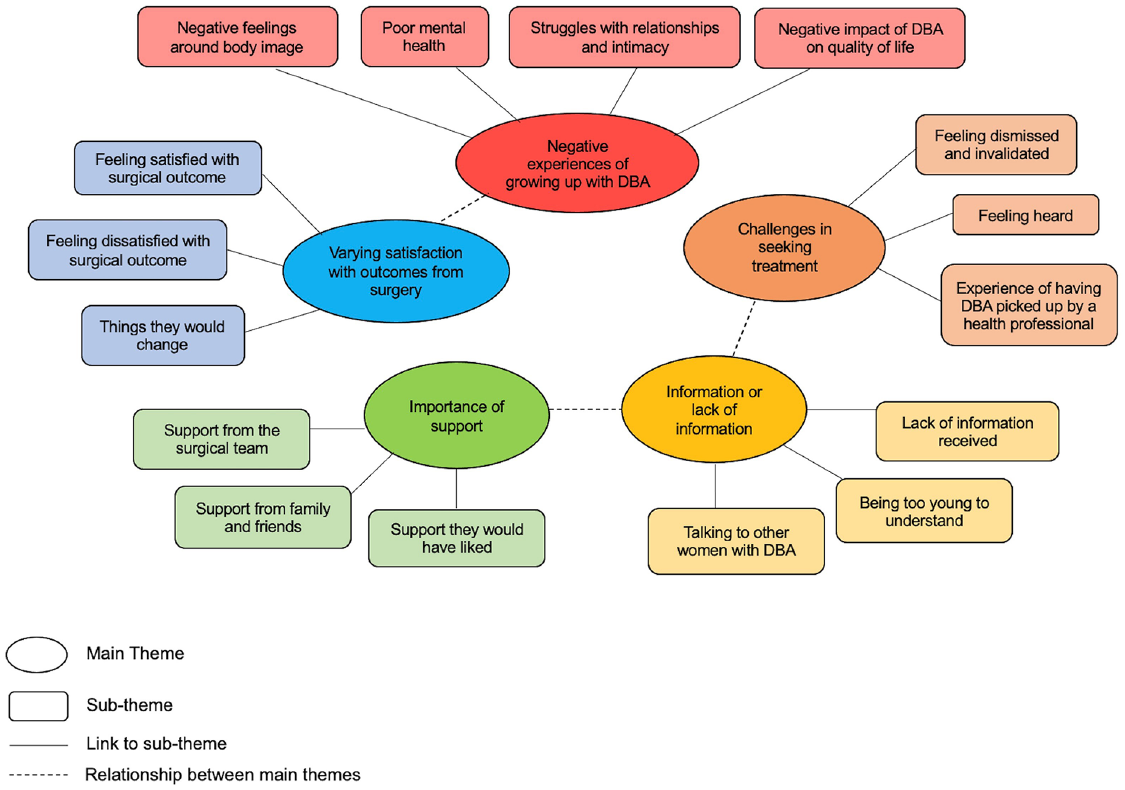
Thematic map of the five main themes and their sub-themes.

After multiple reiterations, five major themes were identified: negative experience of growing up with DBA, challenges seeking treatment, information or lack of information, importance of support and varying satisfaction with outcomes from surgery. Each of the five main themes identified had a number of sub-themes. The relationships between the main and sub-themes as well as any relationship between the main themes are shown in a thematic map, Figure 1. Interview summaries documenting participants’ pre-operative struggles, post-operative outcomes and their main feedback regarding the surgical experience are shown in Supplemental Appendix 2.

### Theme 1: negative experiences of growing up with DBA

#### Negative feelings around body image

All women interviewed identified DBA as being the root cause of their body image issues. Many reported a sense of disconnection and discomfort within their body. For most women, growing up with DBA affected their perceptions of their own bodies and how they compared to others. They found themselves constantly making comparisons to others and wanting to be ‘normal’.

I didn’t feel like a woman, I didn’t feel feminine, I didn’t feel pretty, I felt like a freak. – P7

#### Poor mental health

DBA had varying degrees of impact on mental health amongst participants. Seven women explicitly mentioned poor mental health due to DBA. One woman reported previous suicide attempts. However, it is unclear as to whether this was exclusively due to DBA as she was dealing with other difficult experiences simultaneously. None felt the need to seek mental health help at the time. The remainder of the 14 women acknowledged that DBA may have had some effect on their mental health, without drastic or long-lasting impacts.

It was a long time of me really hating myself and not wanting to be here at all.– P7

There were some days, not like I was suicidal, but I was like my life would be so much easier if I wasn’t around because of the pain it was causing on my shoulder. – P1

Three women also experienced concurrent eating disorders. Although it is unclear as to whether the eating disorders were triggered by DBA, the existence of both did have compounding negative effect on the participants’ mental health.

I was also going through bulimia at the time as well. – P5

#### Struggles with relationships and intimacy

Thirteen out of 14 women noted the effects of DBA on intimacy. For many, this was due to feeling self-conscious, uncomfortable and sexually unattractive which compelled them to hide their breasts.

You can’t wear lingerie because one side doesn’t hold anything. – P2

Six of these women preferred to hide their breasts during intimacy despite their partners being aware of their breast asymmetry.

When it came to my breasts, I did prefer to have them hidden. I figured that if I didn’t like the look of them, no one else would. – P13

#### Negative impact of DBA on quality of life

##### Participation

For most women, DBA mostly affected their participation in sports but not school. Five out of 14 participants were unable to participate in sports as they were unable to find bras with adequate support for their asymmetrical breasts or were self-conscious about the way they looked.

Not having a support bra did hold me back from doing things. – P9

Outside of school participation, 10 out of 14 participants reported negative effects on social participation to some degree.

I would be missing out on social opportunities to protect myself from that embarrassment. – P3

Two women were affected to the point where they socially isolated themselves.

I just really tried to be away from people, and really isolated. – P7

Of note, most women reported challenges in finding the courage to participate in gatherings at the beach and avoided entering the water due to feelings of self-consciousness and fear of others noticing their breasts.

It was just one more thing to be conscious about. Don’t get undressed in front of your friends. Don’t go down to the beach. Don’t do this, don’t do that. – P5

##### Difficulties with clothing

Many women reported being unable to buy the clothes they wanted to wear and wearing baggy outfits to hide their bodies. Feeling uncomfortable in ill-fitting clothes and not feeling sexy were also commonly reported.

Unable to wear nicer and more fitting clothes, did not feel sexy. – P9

I found it really hard to find clothing I was comfortable in. – P13

##### Physical health

Several women also experienced neck, shoulder and back pain due to their DBA.

There were times when I couldn’t move my shoulders more than 15 degrees. – P1

##### Bullying

Almost half the participants experienced some form of bullying due to DBA during their teenage years from both peers and partners.

I moved a couple of schools; friends they would make jokes about it. – P14

Several also received various comments which have become an unforgettable part of their experience.

I was, but only from boyfriends. I had one who I was with for 3 months, he did call me a freak for it which wasn’t fun. – P7

### Theme 2: challenges in seeking treatment

#### Feeling dismissed and invalidated

For most women, their surgical journey began after seeing their general practitioner (GP). Several participants found their GP to be supportive in recognising their DBA as a problem and initiating their surgical journey. For others, their GP experience was a source of frustration where they were told their breasts would catch up or they should lose weight.

My GP still didn’t know what was going on, she kept telling me that it would catch up. . .it was so frustrating. – P2

They would say it’s because you’re fat, because of this, because of that. They never took that into consideration as a problem. . .that was quite frustrating and I didn’t feel heard. – P1

#### Feeling heard

Women who recounted positive GP experiences reported having their DBA recognised and feeling heard. One woman who initially thought she was being insecure felt hopeful after her concerns were recognised by the GP.

I thought I’d go and then they’ll just be like you know, you’re just young and insecure, and then when I went to my GP, she was like no, we can definitely see if we can do something. Even that made such a difference in myself cause I thought there was hope that it could change. – P12

#### Experience of having DBA picked up by a health professional

For five women, their DBA was incidentally recognised after they went to see their GP for other breast-related and unrelated concerns. Three out of five went to see their GP for breast lumps when their DBA was recognised. One was recognised after she sought help for the physical symptoms of DBA and another was generally unwell.

I went to see my doctor for feeling unwell and she asked me to lift my shirt and I just had an absolute meltdown. – P5

### Theme 3: information or lack of information

The information received by participants was sub-categorised into information regarding the surgical procedure and recovery period. Most women felt well informed about the surgical process and what it entailed. Most remembered a good explanation of the risks and benefits, with only 2 out of 14 feeling they received insufficient information regarding risks. Many received information in written form prior to their procedure. They found this beneficial as they were able to refer to it in their own time.

I felt well supported and informed by the surgical team throughout the process. – P13

#### Lack of information received

Some participants were unhappy with the lack of details provided concerning what would specifically happen in the surgery.

They just said that they were doing a reconstruction. Not exactly that they were going to reduce and lift. – P14

Lack of procedural options was noted by six participants; however, many were comfortable with the surgeon’s recommendation.

I was so comfortable with the surgeon and her experience that it wasn’t something I asked about. – P13

One participant would have liked to have been presented with alternative and more natural options outside of surgical procedures.

I guess if she’d known more about options that were out there, even if they weren’t actually good options, she could’ve explained to me that these are options. – P4

#### Being too young to understand

Upon reflection, some participants did note that their suboptimal understanding of the information provided arose from being too young. Of the 14 participants, 5 reported feeling too young to properly understand the long-term implications of the surgery. It was noted that despite the information being presented, the depth of this information was not grasped by some participants.

Especially when you’re younger and you’ve never been through something like that, you don’t know what to expect and I think that was hard. – P11

But I would say maybe wait just a little bit longer so I understood it a little bit more, or even considering the other options that we did have. – P6

One common response was the lack of knowledge around what is ‘normal’ and what their expectations should be at a young age. For a few participants, this led to feelings of being overwhelmed and poorer understanding.

It was overwhelming and like I said, I can’t remember a big chunk of it. – P5

However, it is also worth noting the responses of participants who had their surgical procedures at an older age as they would have liked to have the surgery earlier.

I wish I had the chance to maybe have done it earlier before having children. – P9

#### Talking to other women with DBA

One major consensus amongst most women was the need to talk to others with similar experiences. Some felt that the surgical teams were restricted in terms of the information they could provide as they had not experienced the surgical journey firsthand.

If I had met the people who had the surgery 10 years before I had and actually spoken to them or had them sit in on a consultation, that could have completely altered the whole situation. – P9

One participant was able to speak to a friend who had a similar problem who underwent breast surgery, which she felt was extremely useful.

It was good to have someone that had the same problem give me that information before I went in for surgery for sure. – P10

### Theme 4: importance of support

Most participants described the surgical journey as one full of unknowns and uncertainties, especially in the pre-operative stage. However, participants received support from their friends and family, as well as the medical team, which most of them found important to have throughout the surgical journey.

#### Support from the surgical team

Most of women felt well supported and heard by the surgical team throughout the surgical process.

[The surgeon] was great. She was very straight to the point and I really liked her; they made me feel very comfortable and heard. That really mattered for me, because I was unheard for so long. – P1

Some women also sought help from their GPs during the recovery period, especially when they were unable to get back in touch with the surgical team.

I went to my GP. . . simply because they were easier to get in touch with than the hospital and I didn’t want to clog up the hospital system when I knew I could get good care. – P13

One participant felt that it would be good to be able to seek help from the surgical team during the recovery period rather than the GP.

Yes I think that [talking to the surgical team] would’ve been good as well, because you know they’re the ones that do it. . . whereas your GP is just looking at your scar, he doesn’t actually know what they did. – P2

#### Support from family and friends

In the post-operative stage, most participants reported needing significant support from their friends, partners and families as they struggled with many activities.

I couldn’t drive. I couldn’t laugh or twist my sides. So I couldn’t reach for objects and I couldn’t have sex, and I couldn’t get dressed, like change my own clothes. – P3

Two participants reiterated how having a good support network is key for post-operative recovery and how not having a good support network would have been difficult.

If they took into consideration more that not every patient has a great support circle, I think that would be really good for other young people. – P1

#### Support they would have liked

Participants also gave feedback on areas in which the surgical team could improve on that would have improved their experience. Two participants raised the issue of the lack of long-term follow-up and guidance regarding replacing implants.

No, that’s where it would be really good to have one follow-up person or the person you can talk to if you have any questions. I was young and didn’t really know who to contact. – P1

### Theme 5: varying satisfaction with outcomes from surgery

Amongst the 14 women that were interviewed, there were varied levels of satisfaction with the surgical outcome; 8 women were satisfied, 3 had some reservations, and 3 were unsatisfied.

#### Feeling satisfied with surgical outcome

The eight participants who were satisfied reported they felt ‘normal’ and felt a lot more confident.

Happier, definitely happier, I started to wear t shirts and just wasn’t worrying about whether my prosthetic would fall out, just my quality of life went up; When I get out of the shower and look in the mirror I wouldn’t mind looking. I wouldn’t look at myself and be disgusted or you know.; It’s helped my body image, I feel like I’m normal, you know I don’t feel like a freak, and same for my mental health. – P2

Outcomes participants were most pleased with were having symmetrical breasts which allowed them to wear clothes and bras they wanted, making them feel more confident and empowered. This also allowed them to participate in things they previously did not feel comfortable doing and improved their quality of life and mental health.

I am very pleased with the size and how symmetrical they are now. . . I feel a lot more confident now. . . I used to shop for my boobs and now I can shop for my body as whole which does open my options a lot more. I can get pretty bras and they’re not $100 a pair at minimum. I am very happy, especially with the symmetry. – P1

Two participants emphasised on how the surgery has made them feel ‘whole’ and ‘complete’.

Yeah, and just happy and whole. It made me feel whole. – P2

Participants also talked about their newfound confidence and feeling more comfortable naked and when intimate with their partners.

I would say that I’m happy being completely naked whereas I wasn’t happy to have a shower with [my partner]. . . I would’ve hated it back then. I would do it, but I didn’t feel confident and I think he would feel the difference in me now as well. – P13

One participant experienced complications with her implants resulting in their removal. However, because of the surgical process, the skin around her breasts eventually evened out and she was satisfied with the outcome without needing further reconstruction. However, she was not satisfied with the complications she had to deal with along with scarring that was worse than expected.

When I made the decision to get them out, when she said I could put another brand in, I didn’t want to have to put my body under that again just for another 10 years to do it again. They kind of did its job because it stretched my skin which made [my breasts] even. – P6

However, despite reconstruction surgery and its associated improvements, several participants mentioned how they still experience some mental health and body image issues given how deeply ingrained they are after having dealt with them for such a long time.

Now that it’s all fixed not as much, but even still afterwards you still have that same insecurity cos it’s drilled into your mind. – P12

#### Feeling dissatisfied with surgical outcome

Three participants found that undergoing surgery made no difference for them, and either brought them back to where they were originally or caused new problems. Two of the three participants experienced significant post-operative complications leading to removal of their implants.

I don’t have any prosthetics in now and I said to my surgeon, I can’t be like this for a year because I can’t look at myself, I hate looking at myself now in the mirror, I feel like I’m back to square one. – P2

One participant was not satisfied with how her breasts felt different after her surgery where she only had one implant in one breast, even though she was able to wear clothes and bras that fitted her.

All of a sudden you look and you’re like woah! They’re the same size-ish!. . . I could wear bras that were, you know, fairly comfortable and fitted but then. . . the realisation that your breasts don’t match your body. . . So one was like very very hard, then obviously your natural is very very soft. . .that was something that I found challenging. . . It solves your size problem, but it doesn’t solve how you necessarily look and feel cos you still are odd if that makes sense. – P4

The above participant went on to have her implants removed due to complications and a bilateral breast reduction to even out her breasts. However, she has been left with significantly smaller breasts that are still uneven, which has significantly impacted her relationships and self-confidence.

I still don’t match; I don’t like my breasts; I haven’t been in a relationship since my last surgery, so it’s definitely impacted me in that way, hugely. Yeah confidence levels probably dropped. – P4

She mentioned sometimes regretting undergoing surgery, but was grateful for other improvements like being able to wear clothes and bras that fit.

I have regretted it. . . But then I also know how uncomfortable I was and how I wanted to have a bra that fitted. . . so, yeah, I have at times, but then at other times I’m thankful for it so bit of both. – P4

However, she was cognisant of how her expectations might have undermined the outcome, resulting in her being unsatisfied.

Yes, what was done was asked for, but it hasn’t made me happier about my breasts, no. . . but realistically, the results are as much as the surgeons are able to do which isn’t always perfect. – P4

For one participant, even though surgery corrected the asymmetry and gave her more freedom in clothing choice, she left with more severe scarring than expected, which still makes her self-conscious.

It’s helped with at least reducing the size. . . and the lifting, it’s helped with bringing my nipples higher, so I feel more confident in that way, but I still have all the scars underneath which are the parts that bother me the most. – P14

Another participant developed painful scarring and regretted undergoing surgery.

Yeah, I don’t like the way they feel most of the time. I don’t like the way they look. Even the touch sensitivity [of my scars] sometimes gets really uncomfortable and I just wish that if I had known what I knew now I wouldn’t have done it because that’s just the way my body was intended to be. So it is hard, I look at them and think oh something is a little bit off still. – P5

#### Things they would change

While most participants were pleased with the surgical outcome, several participants voiced minor things they would change if given the opportunity. Regardless, they were still satisfied with the overall outcome and improvement they experienced from the surgery.

##### Tissue scarring

Some participants had more scarring than expected but considered this to be trivial compared to the overall improvement they had.

There’s part of my scarring that is a bit crinkled and they’ve pulled a lot of skin over. . . I don’t really like how that feels because it’s a bit lumpy and the scar is a bit uneven. But I know that it’s between being comfortable and vaguely dislike certain aspects of it or be a misery all day, I would still choose that option and I wouldn’t change my choice. – P1

##### Timing of surgery

Participants who were told to wait until they had their children or until they were older mentioned that they wished they had done the surgery at a younger age.

If I could go back I’d say don’t listen to the GP, she doesn’t know what she’s talking about. – P2

##### Reconstruction options

Some participants report that they wished they had considered different reconstruction options and types or sizes of implants.

I would like probably like to see if there were different options and maybe different ways of how my reconstruction and everything could’ve gone in the first place. – P14

Some participants are considering revision surgery to help them regain their sense of confidence.

I’d actually really like an intimate relationship, so this is why I’ve started thinking about the double implant to help me be confident enough to go out there and look for the opportunity to be in an intimate relationship. – P4

## Discussion

This study explores the experiences of women with DBA who have undergone reconstruction surgery in an Australian healthcare setting to build on the scarcity of qualitative data available on DBA. It has demonstrated the significant pre-operative impacts of DBA on psychosocial well-being and how reconstruction surgery can improve this but is not always a guaranteed solution. Our study has also noted the importance of raising awareness about DBA and providing patients with sufficient information and support throughout the surgical process.

Participants in our study voiced the significant ways in which DBA has negatively impacted their psychosocial well-being in terms of mental health, body image, intimate relationships and overall quality of life. This is similar to findings from Klassen and colleagues where women with breast asymmetry felt uncomfortable in their own skin, described themselves as ‘freaks’ and sought goals of ‘normalcy’ or being like others.^9^

Reconstruction surgery improved psychosocial well-being for the majority of our participants; they reported improved mental health, body image, confidence and overall quality of life. Other studies on reconstruction surgery for breast asymmetry have also demonstrated such improvements post-operatively.^1,6,7,9^ Despite this, over a third of our participants reported persisting body image and mental health issues due to deeply ingrained insecurities. They described this as an inability to get used to the ‘new normal’ or change longstanding habits of avoidance of social activities. This is supported by NiMhurchadha and colleagues who also noted that body image issues that arise during adolescence, a critical stage of development, can cement negative psychosocial behaviours that persist even after corrective surgery.^6^ Despite these ongoing issues, women in our study were still pleased with the outcome and the improvements that they experienced, highlighting that while reconstructive surgery cannot solve all problems, it is still effective in improving overall psychosocial well-being.

In our study, a few participants did not experience post-operative improvements and were dissatisfied because of post-operative complications or unmet expectations. This highlights how reconstruction surgery does not always have positive outcomes. However, we note the correlation between expectation and outcome satisfaction which colleagues NiMhurchadha and Pusic have reported, in that disparities between patients’ expectations and surgical outcomes lead to overall dissatisfaction.^6,18^ NiMhurchadha and colleagues found that women with DBA had higher expectations about being the ‘same’ and ‘perfect’ despite being warned by surgeons to lower their expectations.^6^ Pusic and colleagues also noted that not clarifying outcome expectations can lead to unrealistically high expectations and subsequent overall dissatisfaction with the surgical outcome.^18^ They also highlighted how online information only showcasing ideal post-operative outcomes can contribute to unrealistic expectations.^18^ This raises the need to thoroughly educate patients about the risks and limitations of surgery to help set realistic surgical expectations to prevent post-operative dissatisfaction.

This study is the first to investigate how women sought treatment and the obstacles they faced doing so, specifically in an Australian context. This highlighted the varied experiences women had in seeking treatment for their DBA and the several factors that contribute to this: patients’ fear of having others look at their asymmetrical breasts, as well as patients’ and medical practitioners’ level of awareness about DBA and the surgical treatments available. The negative experiences some participants in this study had when seeking help demonstrated the need to raise awareness within the medical sector to improve the treatment-seeking experience and timely access to treatment. Considering how many of our participants were also unaware of DBA and the treatment available, better public knowledge of breast asymmetry as a developmental condition could also help legitimise seeking treatment. In this aspect, social media can be a powerful tool to raise awareness, especially for younger women.

Our study also delved into participants’ experiences of the surgical journey, in particular the information and support they received. Most women in our study felt well informed and well supported by the surgical team. However, several participants voiced that they would have liked more information. This is similar to Pusic and colleagues’ study, and they recommend that providing more thorough education prior to surgery might be useful.^18^

All participants in our study felt that it would have been useful to have pre-operative support and advice from patients who have undergone the reconstructive process. NiMhurchadha and colleagues have similarly suggested ‘peer support’ pre- and post-operatively to help with body image and confidence.^6^ Support groups are also becoming more commonplace in many surgical and medical fields and have been shown to help patients feel less isolated in their experience of their health conditions and help with health-related decision making.^19^ In the context of our study, such support could help provide more information from a patient perspective prior to surgery and could also address concerns raised post-operatively. It could also provide perspective on expectations surrounding outcomes which can help address the challenge of setting realistic surgical expectations as discussed above. Participants in our study also shared that they were happy to speak to new patients considering symmetrisation surgery.

Of note, some participants in our study raised how their young age contributed to a suboptimal understanding of the information provided. Nuzzi and colleagues have compared post-operative outcomes between younger patients under 18 years of age and older patients undergoing breast reconstruction surgery and found no difference in surgical outcomes, thus concluding that young age or ‘age cut-offs’ should not be a barrier to surgery.^20^ However, there have not been any studies that utilise pre-operative assessments to compare the level of understanding of surgical information provided between younger and older patients undergoing reconstruction surgery. Therefore, young age may be a factor for surgeons to consider when evaluating and educating patients for reconstruction surgery. It is important to note that despite this, participants who reported this issue would not have changed having had the procedure at a young age. Moreover, participants who underwent reconstruction at a later age would have liked to have had procedure at a younger age. This further supports Nuzzi’s findings that corrective surgery, even in young women, can have long-lasting benefits.^20^

Social support from friends and family was also a novel aspect explored in our study, with many participants describing how they needed a lot of support from their family and friends during the recovery period. This also serves as another factor for consideration when evaluating patients for reconstruction surgery to ensure they have sufficient support following such major surgery.

The authors acknowledge that data saturation in qualitative research is not easily defined and may be reached at different points in different studies.^17^ Our study population was relatively homogeneous in that they were all women who underwent surgical correction for DBA. Participants were purposively sampled based on this common criterion, as a result, we would expect saturation of themes to occur sooner than in a more diverse group. Our study also had a semi-structured interview design, making it a fairly narrow area of investigation. Therefore, after conducting 14 interviews with no new codes emerging, the authors felt saturation had been achieved.

A limitation of this study is the potential for recall bias. Many of the participants involved had their reconstruction up to 18 years ago and thus would have to rely on their recollection regarding their experience growing up with DBA, their motivations for surgery and their experience of the surgical process. Another limitation is that interviews conducted in this study were done post-operatively. This precludes the ability to conduct pre-operative assessments to understand patients’ concerns and expectations prior to surgery for comparison to post-operative outcomes. Future research using a prospective approach would allow for pre- and post-operative assessments which can uncover important aspects of reconstructive surgery that are yet to be addressed. This study was also conducted during the COVID-19 pandemic and as a result, restrictions on social distancing and visitors to hospitals were in place for some time. During these times, the researchers conducted interviews using an online platform. Although not ideal, the researchers felt that the platform allowed the participants to stay in their own homes where they would feel safe and comfortable and open to discussing their experiences. A strength of this study is its qualitative approach. This allowed the researchers to document the patient experience in detail rather than report outcomes of a surgical journey based purely on re-admission or complication rates or other measures recorded in hospital.

## Conclusion

This qualitative study has highlighted the subjective experiences and challenges faced by women who have grown up with DBA and undergone symmetrisation surgery for DBA, improving our understanding of the impact of this condition. This study demonstrated improvement in psychosocial well-being post-operatively but highlighted that this is not always the case due to post-operative complications and unmet expectations. This study has also found a need for further education in the public and medical sector on DBA to raise awareness of the condition and its treatment options. Further research in this area is needed to explore the impact of support groups in aiding surgical decision making and also be able to compare pre-operative psychosocial wellness measures to those obtained following surgery.

## Supplemental Material

sj-docx-1-whe-10.1177_17455057241274901 – Supplemental material for A qualitative study on the experiences of women undergoing surgery for developmental breast asymmetry Supplemental material, sj-docx-1-whe-10.1177_17455057241274901 for A qualitative study on the experiences of women undergoing surgery for developmental breast asymmetry by Lorraine Kit Ying Ho, Sahima Jafari, Tamara Crittenden, Phillipa van Essen, Andrea Smallman and Nicola R. Dean in Women’s Health

sj-docx-2-whe-10.1177_17455057241274901 – Supplemental material for A qualitative study on the experiences of women undergoing surgery for developmental breast asymmetry Supplemental material, sj-docx-2-whe-10.1177_17455057241274901 for A qualitative study on the experiences of women undergoing surgery for developmental breast asymmetry by Lorraine Kit Ying Ho, Sahima Jafari, Tamara Crittenden, Phillipa van Essen, Andrea Smallman and Nicola R. Dean in Women’s Health

sj-docx-3-whe-10.1177_17455057241274901 – Supplemental material for A qualitative study on the experiences of women undergoing surgery for developmental breast asymmetry Supplemental material, sj-docx-3-whe-10.1177_17455057241274901 for A qualitative study on the experiences of women undergoing surgery for developmental breast asymmetry by Lorraine Kit Ying Ho, Sahima Jafari, Tamara Crittenden, Phillipa van Essen, Andrea Smallman and Nicola R. Dean in Women’s Health

sj-docx-4-whe-10.1177_17455057241274901 – Supplemental material for A qualitative study on the experiences of women undergoing surgery for developmental breast asymmetry Supplemental material, sj-docx-4-whe-10.1177_17455057241274901 for A qualitative study on the experiences of women undergoing surgery for developmental breast asymmetry by Lorraine Kit Ying Ho, Sahima Jafari, Tamara Crittenden, Phillipa van Essen, Andrea Smallman and Nicola R. Dean in Women’s Health

## Declarations

**Ethics approval:** Ethics approval was obtained from the Southern Adelaide Clinical Human Research Ethics Committee (approval number 98.14).

**Consent to participate:** All participants were provided with a Participant Information Sheet and informed consent was obtained prior to any participation. Written informed consent was obtained from participants who had face-to-face interviews, and informed consent was obtained verbally from participants who had online interviews which were audio-recorded prior to the interview. Participants were informed in the information sheet that anonymised research findings may be published and informed consent for publication was given based on this understanding. Informed consent for publication was therefore provided by all participants as part of the consent to participate process.

**Availability of data and materials:** Not applicable.
