# Supplementary material for: A qualitative study on the experiences of women undergoing surgery for developmental breast asymmetry
Source: Womens Health (Lond). 2024 Sep 5;20:17455057241274901. doi: 10.1177/17455057241274901 (PMC11378208; doi:10.1177/17455057241274901)
Supplement: sj-docx-2-whe-10.1177_17455057241274901 – Supplemental material for A qualitative study on the experiences of women undergoing surgery for developmental breast asymmetry [file sj-docx-2-whe-10.1177_17455057241274901.docx]

# Supplementary Appendix 1. Interview Question Guide for Qualitative Interviews

**Experiences living with breast asymmetry**

1. How old were you when your breasts started to develop?

Optional prompts:

- Were you still in primary school?
- When did you first start to notice the difference in your breasts?
- Who did you first talk to about it?

1. Were you bullied or made to feel uncomfortable about your breasts at school?

Optional prompts:

- Do you think the problem with your breasts affected your participation at school?
- In sport?
- In other areas?

1. Do you think the problem with your breasts has affected your confidence?

Optional prompts:

- Has it affected you in forming relationships?
- If you are in a relationship, do you think that your breast problem impacts on how you feel or act when you are intimate with your partner?
- Do you / did you tend to try to hide your breasts from your partner?
- Do you think DBA has affected your body image?
- Do you think DBA has impacted your sexuality growing up?
- How has it affected your mental health?

1. Overall do you think the problem with your breasts not matching has had much of an impact on your health or quality of life?

**Surgery and the reconstruction process**.

1. How did you seek treatment?

Optional prompts:

- When did you first seek treatment?
- Who told you that surgical options were available?
- Did you speak to your family, partner or friends before deciding to seek treatment?
- How long did it take before your reconstructive surgery began?
- Were there any obstacles in particular that you found difficult prior to your surgery? (e.g. financial, emotional concerns)

1. What procedures have you undergone?

Optional prompts:

- Where and when did the procedures take place?
- How did you feel about having surgery before it took place?
- Were there any complications? How were these managed?
- Will you have further procedures for completion of the reconstruction?

1. How long was your recovery stage?

Optional prompts:

- How long did you stay in hospital?
- How long did you need to take off work/studies?
- What were you unable to do during your recovery process?
- How were you followed up?
- Did you know who to contact if you had concerns at this time?

1. Did the surgical team adequately support you and inform you throughout the process?

Optional prompts:

- What information did you receive about the process of surgery?
- Did you have options in regards to different types of breast reconstruction? If so, why did you choose one option over another?
- Were the risks and benefits explained to you? In a way that you understood?
- Were you told how much pain to expect? Did you think this was accurate?
- Were you given information about healing and recovery time?
- Were you told what to expect in terms of scarring?
- Who did you talk to if you had questions or concerns?
- Is there anything you felt was not adequately explained, or could have been explained better?
- Did you feel well supported by family and friends during the process? What additional support did they provide?
- Did your family and friends feel supported during the process?

**Outcome of your surgery**

1. Are you satisfied overall with the outcome of the surgery?

Optional prompts:

- Is the outcome better, equal to, or worse than you expected?
- What outcome are you particularly pleased with?
- What would you change if given the opportunity?
- If you have a partner, what do they think of the outcome?

1. Were you satisfied overall with the surgical process and team?

Optional prompts:

- Were you given enough information and support by the team?
- Do you think the surgical team was experienced and well equipped in treating you?
- Is there any way in which the team could have improved your experience?
- Is there any other way in which your experience could have been improved?

1. How has the reconstruction impacted you?

Optional prompts:

- - - - Has your confidence changed?
      - Have your relationships or intimacy with your partner changed?
      - Overall, how has your life changed since having the surgery?
      - Has the surgery improved your mental health and/or body image?

1. Overall, what was done particularly well in your experience?
2. Overall, what could be improved?
3. Would you encourage others in your position to have the procedure?
4. What advice would you give others?
5. Is there anything else you would like to add that we have not covered today?
