# Supplementary material for: A qualitative study on the experiences of women undergoing surgery for developmental breast asymmetry
Source: Womens Health (Lond). 2024 Sep 5;20:17455057241274901. doi: 10.1177/17455057241274901 (PMC11378208; doi:10.1177/17455057241274901)
Supplement: sj-docx-3-whe-10.1177_17455057241274901 – Supplemental material for A qualitative study on the experiences of women undergoing surgery for developmental breast asymmetry [file sj-docx-3-whe-10.1177_17455057241274901.docx]

# Supplementary Appendix 2. Interview Summary Table

*Colours in the ‘Surgical Outcome’ column represent overall satisfaction with surgical outcome. Green = satisfied; orange = some discontentment; red = dissatisfaction.*

| Participant | Pre-Operative Struggles | Surgical Outcome | Comments about Surgical Journey |
| --- | --- | --- | --- |
| P1 | Poor body image and self-esteem  Mental health affected  Experienced physical pain due to DBA which affected social, school and sports participation  Wore baggy clothes to hide breasts    Unable to find suitable bras and clothes | Satisfied with size and symmetry    No longer has physical pain, able to participate in the things that she wants to do    Able to wear bras and clothes that fit properly, increased clothing options    Improved confidence and body image | Dismissed and invalidated by GP  Satisfied with surgical team    Would have liked to talk to someone about the surgical process and get more information as she felt underprepared for it |
| P2 | Bullied for DBA  Poor body image and self-esteem  Avoided swimming and going to the beach    Played sports but wore multiple layers to hide DBA    Used silicone inserts and shoulder pads to fill bras | Initially satisfied with size, symmetry and no longer needing to hide her breasts after the first surgery    Improved confidence and clothing choice which made her feel more empowered    Back to original state after having breast implants removed due to post-operative complications | Dismissed and invalidated by GP  Felt well supported and informed by surgical team    Wish she had undergone reconstruction at a younger age instead of waiting till she had children |
| P3 | Bullied for DBA  Poor body image and self-esteem    Avoided participating in school, sports and social events | Satisfied with size, symmetry, and being able to fit into clothes and bras    Improved confidence, body image, and intimacy    Feels whole and complete    Able to participate in more social events | DBA recognised while being assessed by GP for other breast concerns  Felt well supported and informed by surgical team  Would have liked more information about implants    Would have liked to talk to other women with DBA who have undergone reconstruction |
| P4 | Poor body image and self esteem, felt depressed    Relationships and intimacy severely affected  Hid breasts when playing sports    Unable to find comfortable clothes and bras  Social participation affected, avoided swimming/going to the beach | Did not like how both breasts did not look and feel the same due to only one breast having an implant    Able to find comfortable bras and clothes that fit    Implant removed due to post-operative complications, breasts still asymmetrical    Relationships and intimacy still severely affected post-reconstruction | DBA recognised while being assessed by GP for other breast concerns  Felt supported by surgical team    Would’ve liked to have surgical options (both suitable and non-suitable) explained to her  Has had moments where she regretted having reconstruction due to unideal outcomes and complications    Would have liked to talk to other women with DBA who have undergone reconstruction |
| P5 | Self-conscious and hid breasts during intimacy    Did not feel sexually attractive  Dealt with other mental health problems at the same time    Avoided swimming and going to the beach | Not satisfied with surgical outcome due to breasts still being asymmetrical, nipples being uneven, and painful/sensitive scarring that was worse than expected    Thinking of removing implants    Regrets doing the surgery    Increased clothing options and feels more confident being able to wear more clothes, but still unsatisfied with breasts | DBA recognised while being assessed by GP for non-breast related concerns  Would have liked to explore more options    Felt that she was too young to have thought about what to ask during pre-op appointments    Felt well supported and informed by the surgical team    Would have liked to talk to other women with DBA who have undergone reconstruction |
| P6 | Self-conscious about breasts    Body image and mental health not severely affected  Hid breasts during intimacy | Initially satisfied with symmetry after inserting implants however scarring worse than expected  Experienced complications with implants resulting in removal of implants, but breasts became symmetrical due to skin stretching    Increased clothing options which improved confidence  Ongoing long-lasting self-consciousness after having grown up with it for so long | DBA recognised while being assessed by specialist for non-breast related concerns  Satisfied with support and information from surgical team    Would have liked to have waited until she was a bit older to do the procedure so she could understand the process better |
| P7 | Bullied for DBA  DBA added to other significant mental health problems when growing up    School, social and sports participation severely affected, isolated herself  Relationships and intimacy severely affected  Self-conscious during intimacy | Satisfied with size, symmetry and how natural they feel    Feeling happy and more confident    Able to participate in more social and sports activities  Increased clothing options | GP acknowledged concerns about DBA  Felt well supported and informed by Nurse Practitioner and surgical team    Would have liked to talk to someone with DBA who had gone through the reconstruction process |
| P8 | Embarrassed by DBA, did not have confidence to seek help from doctors    Able to participate in school, social and sports events but always hid breasts by wearing baggy clothes and/or using silicone bra inserts    Did not dare to enter a relationship | Reconstruction not complete but satisfied with symmetry  Still doesn’t wear tight or low-cut clothing due to long-standing self-consciousness    Feels that she’s become more confident and stronger as a person | DBA recognised while being assessed by GP for other breast concerns  Felt well supported and informed by surgical team    Wish she sought help for DBA when she was younger |
| P9 | Embarrassed by DBA, but felt that mental health was not greatly affected  Hid breasts by wearing baggy clothes and stuffing bra    Did not feel sexually attractive  Unable to find suitable bras and clothes    Had physical pain due to DBA which affected sports participation | Satisfied with size and symmetry    Increased bra clothing options and improved confidence    Improved body image | GP acknowledged concerns about DBA  Would have liked to have had reconstruction done earlier before having children    Felt well supported and informed by the surgical team |
| P10 | Self-conscious about DBA    Embarrassed about DBA when it came to relationships    Hid breasts by wearing multiple layers, hid breasts when intimate    Unable to find suitable bras and clothes  Experienced physical pain due to DBA  Avoided participating in sports due to pain and lack of adequate bra support | Satisfied with shape, size and symmetry    Increased bra and clothing options    Improved body image, mental health and confidence    No longer experiences physical pain, able to exercise | Dismissed and invalidated by GP  Felt that it was useful having the opportunity to talk to a friend who had undergone breast surgery to learn more about the procedure    Felt well supported and informed by surgical team |
| P11 | Embarrassed and self-conscious about DBA    Felt that it affected her quality of life socially and psychologically    DBA contributed to existing mental health problems  Hid breasts by stuffing bra with tissues and silicone inserts    Relationships impacted  Unable to find comfortable bras    Avoided swimming and beach outings | Satisfied initially after surgery, however breasts have become asymmetrical again due to breast feeding and changes in weight    Improved confidence  Increased clothing options | Dismissed and invalidated by GP  Felt well supported and informed by surgical team    Felt that things were explained very well when she was having surgery at such a young age    Would have liked more information about longevity of implants, risks of having implants in for so long, and when to revisit the issue    Would have liked long-term follow up and support  Would have liked to talk to other women with DBA who had undergone a similar procedure |
| P12 | Body image and mental health affected negatively  Limitations in clothing choice  Relationships affected  Tried to hide by stuffing bra with tissues  Difficulty engaging in physical activity, avoided swimming | Satisfied with size and mastopexy  Improved body image, mental health and confidence, however still has some long-standing self-consciousness  Increased clothing options  Improved social participation and overall quality of life | GP acknowledged concerns about DBA  Felt well supported by surgical team, felt understood and recognised |
| P13 | Poor body image and self-esteem  Intimacy affected, preferred having bra on or hiding breasts  Unable to find comfortable clothing, limited clothing options  Social participation affected, avoided swimming/going to the beach | Outcome better than expected, satisfied with shape    Increased bra and clothing options  Improved body image, confidence and quality of life | GP acknowledged concerns about DBA  Felt well supported and informed by the surgical team throughout process    Felt well supported by GP during recovery period |
| P14 | Bullied for DBA    Anxious and insecure due to DBA    Felt uncomfortable with physical activities    Used silicone bra inserts to hide DBA    . | Not satisfied with outcome as scarring worse than expected, continues to feel anxious and insecure about breasts due to scarring    Pleased with having more clothing options and not having to hide breasts | GP acknowledged concerns about DBA  Did not feel well informed about surgical process  Did not feel well informed regarding recovery period    Did not feel well supported once post-op follow-ups were done  Would have preferred longer-term follow up |
