# Supplementary material for: A qualitative study on the experiences of women undergoing surgery for developmental breast asymmetry
Source: Womens Health (Lond). 2024 Sep 5;20:17455057241274901. doi: 10.1177/17455057241274901 (PMC11378208; doi:10.1177/17455057241274901)
Supplement: sj-docx-4-whe-10.1177_17455057241274901 – Supplemental material for A qualitative study on the experiences of women undergoing surgery for developmental breast asymmetry [file sj-docx-4-whe-10.1177_17455057241274901.docx]

# Supplementary Material – Titles and Descriptions

**Title:**

Supplementary Appendix 1. Interview Question Guide for Qualitative Interviews

**Description:**

Interview question guide used for conducting qualitative interviews in the article “A qualitative study on the experiences of women undergoing surgery for developmental breast asymmetry” by Ho LKY, Jafari S, Crittenden T, van Essen P, Smallman A and Dean NR in Women’s Health Sage Journals.

**Title:**

Supplementary Appendix 2. Interview Summary Table

**Description:**

A summary table documenting the 14 qualitative interviews conducted in the article “A qualitative study on the experiences of women undergoing surgery for developmental breast asymmetry” by Ho LKY, Jafari S, Crittenden T, van Essen P, Smallman A and Dean NR in Women’s Health Sage Journals.
